# Testing the Feasibility and Usability of a Novel Smartphone-Based Self-Management Support System for Dialysis Patients: A Pilot Study

**DOI:** 10.2196/resprot.7105

**Published:** 2017-04-20

**Authors:** Aki Hayashi, Satoko Yamaguchi, Kayo Waki, Katsuhito Fujiu, Norio Hanafusa, Takahiro Nishi, Hyoe Tomita, Haruka Kobayashi, Hideo Fujita, Takashi Kadowaki, Masaomi Nangaku, Kazuhiko Ohe

**Affiliations:** ^1^ Department of Ubiquitous Health Informatics Graduate School of Medicine The University of Tokyo Tokyo Japan; ^2^ Department of Diabetes and Metabolic Diseases Graduate School of Medicine The University of Tokyo Tokyo Japan; ^3^ Department of Cardiovascular Medicine Graduate School of Medicine The University of Tokyo Tokyo Japan; ^4^ Division of Total Renal Care Medicine The University of Tokyo Hospital Tokyo Japan; ^5^ Department of Blood Purification Kidney Center Tokyo Women's Medical University Tokyo Japan; ^6^ Nishi Clinic Tokyo Japan; ^7^ Yuujin Clinic Tokyo Japan; ^8^ Business Development of Healthcare Business Smart-Life Solutions Department NTT DOCOMO, Inc Tokyo Japan; ^9^ Division of Nephrology and Endocrinology Graduate School of Medicine The University of Tokyo Tokyo Japan; ^10^ Department of Biomedical Informatics Graduate School of Medicine The University of Tokyo Tokyo Japan

**Keywords:** telemedicine, mobile phone app, hemodialysis, self-management

## Abstract

**Background:**

Diet and fluid restrictions that need continuous self-management are among the most difficult aspects of dialysis treatment. Smartphone applications may be useful for supporting self-management.

**Objective:**

Our objective is to investigate the feasibility and usability of a novel smartphone-based self-management support system for dialysis patients.

**Methods:**

We developed the Self-Management and Recording System for Dialysis (SMART-D), which supports self-monitoring of three mortality-related factors that can be modified by lifestyle: interdialytic weight gain and predialysis serum potassium and phosphorus concentrations. Data is displayed graphically, with all data evaluated automatically to determine whether they achieve the values suggested by the Japanese Society for Dialysis Therapy guidelines. In a pilot study, 9 dialysis patients used SMART-D system for 2 weeks. A total of 7 of them completed questionnaires rating their assessment of SMART-D’s usability and their satisfaction with the system. In addition, the Kidney Disease Quality of Life scale was compared before and after the study period.

**Results:**

All 9 participants were able to use SMART-D with no major problems. Completion rates for body weight, pre- and postdialysis weight, and serum potassium and phosphorus concentrations were, respectively, 89% (SD 23), 95% (SD 7), and 78% (SD 44). Of the 7 participants who completed the usability survey, all were motivated by the sense of security derived from using the system, and 6 of the 7 (86%) reported that using SMART-D helped improve their lifestyle and self-management.

**Conclusions:**

Using SMART-D was feasible, and the system was well regarded by patients. Further study with larger scale cohorts and longer study and follow-up periods is needed to evaluate the effects of SMART-D on clinical outcomes and quality of life.

## Introduction

Management of fluids, sodium, serum potassium, and phosphorus is critical in dialysis therapy. Excessive amounts of any of these may lead to dialysis-related complications such as heart failure, which has been identified as one of the risk factors in dialysis for mortality [1-3]. As a consequence, patients on dialysis are prescribed a diet therapy that restricts intake of sodium, potassium, phosphorus, and fluids. Poor adherence to that diet increases the risk of progression of hypertension and cardiovascular disease, eventually resulting in heart failure, which increases the mortality rate [2,4-6].

Interdialytic weight gain (IWG)—which is measured as (current predialysis weight – previous postdialysis weight)/dry weight × 100—is an indicator of patient adherence to diet therapy, reflecting their sodium and fluid intake. It is also important in itself because IWG is associated with blood pressure and mortality [1,5]. Moreover, increase in percentage of IWG is associated with an increase in predialysis systolic blood pressure [4].

Serum potassium and phosphorus levels are also good indicators of dialysis patient adherence to a diet therapy, and they are also associated with mortality. A recent study reported that higher potassium intake is associated with increased death risk [7]. Similarly, looking at mineral values, phosphorus level is considered the strongest predictor of mortality for dialysis patients [3,8]. Accordingly, dialysis patients are advised to restrict protein intake and avoid processed foods.

That is easier said than done. Patients receiving hemodialysis often display great difficulty in adhering to a diet therapy that requires strict and continued self-management over a long period of time. The very mechanics of self-management make adherence difficult. Some patients record their body weight and dietary content in a paper diary. But recording data that way is unrewarding because it does not let patients know whether they are achieving target values or let them easily track changes in the data. Systems that do facilitate such understanding of patients’ current status and changes in it may motivate patients, leading to better self-management with improved adherence to diet therapy and improved survival.

Improving quality of life (QOL) is also crucial for dialysis patients because health-related QOL as measured by the Kidney Disease Quality of Life Short Form (KDQOL-SF)[9,10] was shown to be a predictor of mortality and hospitalization in dialysis patients, even after taking into account other risk factors [11,12]. Improving self-management is an effective way to improve QOL for hemodialysis patients [13-15], raising the expectations that tools to help self-management may be effective in improving their QOL.

It was obvious that recent developments in mobile technology—such as smartphone and tablet computer apps—could be the basis for developing a platform for systems to improve self-management of chronic disease. We developed DialBetics, a novel smartphone-based self-management support system for type 2 diabetes patients that provides those patients with real-time advice on diet and lifestyle based on the patients’ at-home measurements and input [16]. The system significantly improved glycemic control by helping patients improve self-management skills.

Given the importance of self-management for dialysis patients, similar self-management support systems are likely to be effective, and, indeed, there have been a few reports of success with self-management support systems for dialysis patients based on mobile technology. Sevick et al [17] reported a case in which a dietary self-monitoring system based on a personal digital assistant (PDA) device augmented by a dietician’s behavioral counseling was effective in reducing IWG and alleviating hyperphosphatemia and hyperkalemia. Connelly et al [18] reported the effect of a mobile app for hemodialysis patients that assisted them in recording their dietary intake and showed them their consumption levels of fluid, sodium, and potassium: 12 of 18 of those patients said that the app helped them modify their diet.

Although these reports are encouraging, the systems involved are relatively complicated and painstaking; patients must faithfully enter every detail of their daily meals. To help improve adherence by hemodialysis patients in the long term, we felt that a simpler system might be preferable.

To that end, we developed the Self-Management and Recording System for Dialysis (SMART-D), a simple smartphone-based system focused on three key factors—IWG, potassium, and phosphorus—that are associated with mortality but are modifiable by improving diet. SMART-D helps patients record body weight, predialysis serum potassium, and phosphorus concentrations with reference to target values with all recorded data displayed in a line chart so patients can recognize at a glance any changes and see whether they are meeting those target values. We conducted a pilot study to evaluate the feasibility and usability of the system for patients at two dialysis facilities in Tokyo.

## Methods

### Design of the Self-Management and Recording System for Dialysis

**Figure 1:**
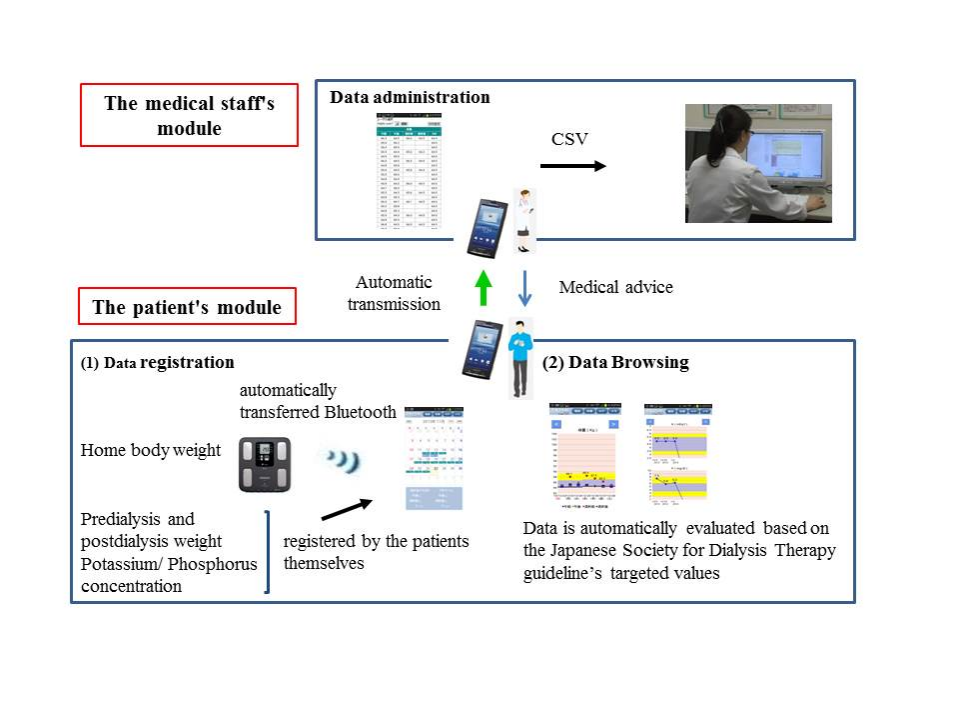
An overview of the Self-Management and Recording System for Dialysis.

**Figure 2:**
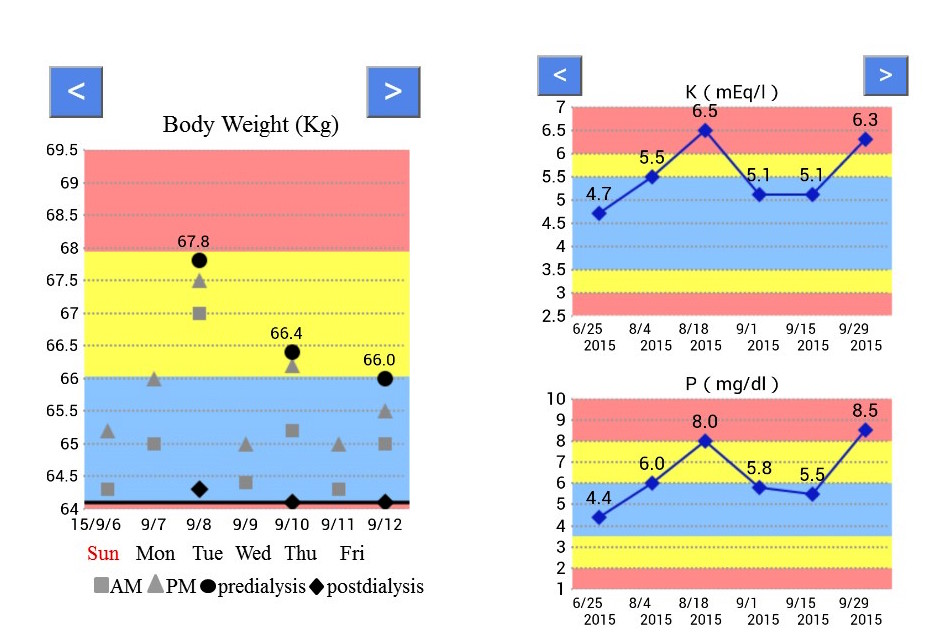
Sample view of the Self-Management and Recording System for Dialysis screen.

The system is composed of two modules (Figure 1). The first—the data management and evaluation module—is for entry of patients’ body weight, serum potassium, and phosphorus concentrations. Body weight (measured twice a day, in the morning and the afternoon) is either automatically transferred by Bluetooth from the weight scale to the patients’ smartphone or entered manually by the patients. When entered manually, if the weight does not seem consonant with the patient’s other recent entries—if it is unreasonably high or low—the smartphone gives an alert to notify the patient of a possible inputting error. Patients manually enter their predialysis serum potassium and phosphorus concentrations; these come from blood tests at their clinic visits (generally every 2 weeks) where they usually receive the full results of the tests. All the data are automatically evaluated according to target values and displayed as a graph so patients can track the day-to-day trend of their weight and the general trend of their potassium and phosphorus levels. Body weight, IWG, and serum phosphorus concentrations are evaluated based on the Japanese Society for Dialysis Therapy (JSDT) guidelines; serum potassium concentrations are evaluated based on annual statistics provided by the JSDT. Optimally, IWG is <3% of the dry weight (DW) when the interval between dialysis sessions is one day and <6% of the DW when the interval is 2 days; serum potassium concentrations are ≥3.5 mEq/L and ≤5.5 mEq/L; serum phosphorus concentrations are ≥3.5 mg/dL and ≤6.0 mg/dL (Figure 2). To help patients monitor their status at a glance, the background of the graph is color-coded: blue if within the range of target values, yellow if marginally outside, and red if seriously outside the target values for body weight (specifically, blue if ≥100% and <103% of DW, yellow if ≥103% and <106% of DW, red if <100% or ≥106% of DW; for potassium, blue if ≥3.5 and ≤5.5 mEq/L, yellow if ≥3.0 and <3.5/>5.5 and <6.0 mEq/L, red if <3.0 or >6.0 mEq/L; and for phosphorus, blue if ≥3.5 and ≤6.0 mg/dL, yellow if ≥2.0 and <3.5/>6.0 and ≤8.0 mg/dL, red if <2.0 or >8.0 mg/dL).

All data are automatically sent by the smartphone to the medical staff administrator module, SMART-D’s second module, which allows the medical staff in dialysis facilities to monitor the data transferred from each patient’s data management and evaluation module and give advice on intake if the readings suggest that intervention is advisable. Patient use of the system is output by a comma-separated values (CSV) file, so how often patients input data and how often they checked their data can also be monitored.

### Study Design and Participants

A questionnaire survey about QOL and a 2-week pilot study whose purpose was to evaluate the feasibility and usability of the system were designed and approved by the Institutional Review Board of the University of Tokyo Hospital. Participants were recruited at two outpatient hemodialysis facilities in Tokyo, with written informed consent obtained from all. To be eligible as participants, patients had to have end-stage renal disease—having received outpatient hemodialysis treatment for at least 2 years—and be aged 20 years or older with an average IWG rate >5% of DW and serum potassium concentration >5.5 mEq/L or serum phosphorus concentration >6 mg/dL when the interval between dialysis sessions was 2 days. Patients who proved unable to use a smartphone correctly because of dementia, vision disorder, or other reasons were excluded. A total of 20 patients agreed to participate in the survey; of those, 9 who were willing to participate in the pilot study using SMART-D were assigned to the SMART-D group. A total of 11 patients who did not wish to use SMART-D but agreed to give their clinical data (IWG, serum potassium, and phosphorus concentration) and to complete the survey were assigned to the non–SMART-D group. All participants would continue their regular dialysis treatment 3 times a week. There were roughly the same number of each group at each dialysis facility. However, those using SMART-D at one dialysis facility used it for 2 weeks from March to April 2014, and those at the other dialysis facility used SMART-D for 2 weeks from September to October 2014. The IWG, serum potassium, and phosphorus concentrations at baseline and follow-up periods were taken, respectively, 2 weeks before and after the study period.

**Figure 3:**
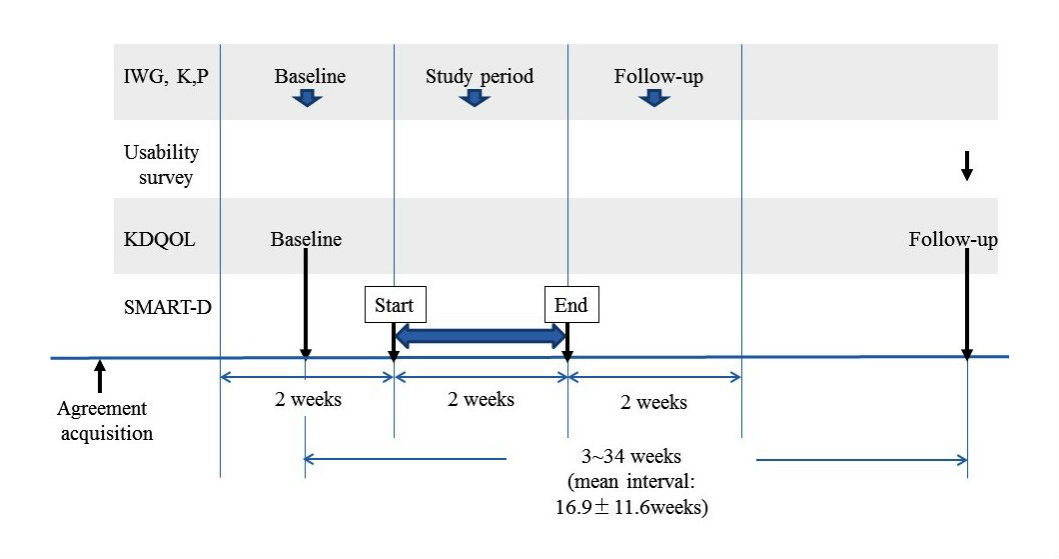
Timeline of the study.

The research team included a nephrologist and system administration specialists, experts in technical and database apps, and a research nurse. The questionnaire survey that patients completed was the Japanese version of the questionnaire summary of dialysis self-care activities and the KDQOL-SF version 1.3 [19]; the survey was made both before and after the study period (the mean interval was 16.9 [SD 11.6] weeks) (Figure 3). One participant in the SMART-D group was hospitalized and one in the non–SMART-D group changed dialysis facilities before completion of the KDQOL during follow-up. This means that only 8 SMART-D users and 10 non–SMART-D users completed the Japanese version of the KDQOL after the study period.

For each participant in the SMART-D group, frequency of the SMART-D use was evaluated by completion rates for body weight in the morning and the afternoon, predialysis and postdialysis weight, and serum potassium and phosphorus concentrations. Completion rates of body weight in the morning and the afternoon, predialysis and postdialysis weight and serum potassium and phosphorus concentrations were calculated as (1) number of entries/(number of days × 2) × 100, (2) number of entries/(number of dialysis days × 2) × 100, and (3) number of entries/number of blood-test days × 100.

Questionnaires rating participant assessment of SMART-D’s usability and their satisfaction with the system were collected after the study ended (Figure 3). Of the 9 SMART-D users, one was hospitalized as mentioned above and one did not complete the usability survey because that participant dropped out before completion of the survey, so only 7 SMART-D users completed the questionnaires.

### Study Methods

Participants in the SMART-D group received a smartphone (Samsung GALAXY Note II SC-02E) and the scale paired with the smartphone (Omron HBF-206IT). In this study, body weight of SMART-D users was measured at home every day and automatically transmitted to the smartphone. On dialysis days, the SMART-D group measured their body weight before and after dialysis. They also entered their predialysis blood test results. Participants in the non–SMART-D group followed their usual self-care regimen.

Members of the SMART-D group were asked to contact the research team nurse by smartphone or email only for technical questions or if, for some reason, they got a weight-input error alert. For questions related to their health status, they were asked to consult their primary physicians or the staff at the dialysis facility.

### Statistical Analysis

Paired *t* tests or Wilcoxon tests were performed to compare IWG, serum potassium concentrations, serum phosphorous concentrations, and KDQOL before and after the study period. Unpaired *t* tests and Fisher exact tests were performed to compare baseline characteristics of the two groups. Repeated measures analysis of variance was performed to compare IWG, serum potassium, and phosphorus concentrations at baseline, study period, and follow-up period. We considered *P* values <.05 statistically significant. All statistical analyses were performed with Excel (Microsoft Corp) or EZR (Saitama Medical Center) [20].

## Results

### Baseline Characteristics

Demographic characteristics of the 9 participants in the SMART-D group are shown in Table 1. The mean age was 47.9 (SD 14.4) years, with the average dialysis vintage being 6.0 (SD 2.9) years. All participants had been receiving dialysis 3 times a week. Their average IWG was 2.2% (SD 0.4) DW/day, average hemoglobin level 10.8 (SD 0.6) g/dL, serum albumin concentration 3.7 (SD 0.2) g/dL, serum potassium concentration 4.9 (SD 0.6) mEq/L, and serum phosphorus concentration 5.4 (SD 1.2) mg/dL. The average number of body weight measurements per day was 1.2 (SD 1.3). Of 9 participants, 7 (78%) were users of a smartphone, PDA, and the Internet, while 2 (22%) were users of a feature phone and nonusers of the Internet (Table 1).

Their demographic characteristics were compared with those of the non–SMART-D group (n=11) who did not wish to use SMART-D. Patients in the non–SMART-D group were older (60.3 [SD 10.5] years, *P*=.04). Of 11 participants, only 3 (27%) were smartphone and PDA users and 4 (36%) were Internet users (Table 1).

**Table 1:** Baseline characteristics of the participants in the study.

			Total (n=20)	SMART-D^a^ (n=9)	Non–SMART-D (n=11)	*P* value^b^
**Demographics**						
	**Sex, n (%)**					
		Male	13 (65)	6 (67)	7 (64)	>.99
		Female	7 (35)	3 (33)	4 (36)	
	Age (years), mean (SD)		54.7 (13.6)	47.9 (14.4)	60.3 (10.5)	.04
**Medical information**								
	Dialysis vintage (years), mean (SD)		8.6 (5.6)	6.0 (2.9)	10.7 (6.7)	.15
	**Hemodialysis frequency in a week, n (%)**					>.99
		Three times	20 (100)	9 (100)	11 (100)	
	**Primary cause of end stage renal disease, n (%)**					.20
		Diabetes mellitus	6 (30)	3 (33)	3 (27)	
		Glomerulonephritis	4 (20)	0 (0)	4 (36)	
		Nephrosclerosis	4 (20)	3 (33)	1 (9)	
		Malignant hypertension	1 (5)	1 (11)	0 (0)	
		Polycystic kidney disease	1 (5)	0 (0)	1 (9)	
		Rheumatoid arthritis	1 (5)	0 (0)	1 (9)	
		Unknown	3 (15)	2 (22)	1 (9)	
	**Comorbid conditions, n (%)**					.85
		Diabetes	5 (25)	2 (22)	3 (27)	
		Hypertension	15 (75)	8 (89)	7 (64)	
		Lipid disorders	2 (10)	1 (11)	1 (9)	
		Cardiovascular disease	2 (10)	2 (22)	0 (0)	
		Cerebrovascular disease	1 (5)	1 (11)	0 (0)	
**Disease status**						
	Predialysis weight (kg), mean (SD)		69.5 (17.8)	76.4 (20.3)	63.8 (14.0)	.12
	IWG^c^(%DW^d^/day), mean (SD)		2.2 (0.4)	2.2 (0.4)	2.2 (0.4)	.92
	Hemoglobin (g/dL), mean (SD)		11.4 (1.5)	10.8 (0.6)	11.9 (1.8)	.048
	Serum albumin concentrations (g/dL), mean (SD)		3.8 (0.2)	3.7 (0.2)	3.8 (0.2)	.24
	Serum potassium concentrations (mEq/L), mean (SD)		5.0 (0.7)	4.9 (0.6)	5.1 (0.8)	.40
	Serum phosphorus concentrations (mg/dL), mean (SD)		5.2 (1.1)	5.4 (1.2)	5.0 (1.1)	.41
**Lifestyle**						
	Weight measurement frequency per day, mean (SD)		0.9 (1.0)	1.2 (1.3)	0.6 (0.7)	.32
	**Cell phone user, n (%)**					.07
		Feature phone	9 (45)	2 (22)	7 (64)	
		Smartphone	10 (50)	7 (78)	3 (27)	
		Non–cell phone user	1 (5)	0 (0)	1 (9)	
	Portable digital assistant user, n (%)		10 (50)	7 (78)	3 (27)	.07
	Internet user, n (%)		11 (55)	7 (78)	4 (36)	.09

^a^SMART-D: Self-Management and Recording System for Dialysis.

^b^*P* value between the SMART-D group and the non–SMART-D group.

^c^IWG: interdialysis weight gain.

^d^DW: dry weight.

### Feasibility and Usability

All 9 patients in the SMART-D group were able to complete the 2-week use of the system without any major problems. The mean number of daily entries for the dialysis date when there were 4 items to be entered (body weight in the morning and afternoon, predialysis weight, and postdialysis weight) was 3.9 (SD 0.2). The mean number of daily entries for the nondialysis date when there were 2 items to be entered (body weight in the morning and afternoon) was 1.8 (SD 0.5). The average completion rates for body weight in the morning and the afternoon and predialysis/postdialysis weight were, respectively, 89% (SD 23) and 95% (SD 7). The average completion rate for serum potassium and phosphorus concentrations was 78% (SD 44) (Table 2).

**Table 2:** Average number of daily entries and completion rates (n=9).

		Mean (SD)
**Mean number of daily entries**		
	Dialysis date (4 items to be entered)	3.9 (0.2)
	Nondialysis date (2 items to be entered)	1.8 (0.5)
**Completion rate (%)**		
	Body weight in the morning and the afternoon	89 (23)
	Predialysis/postdialysis weight	95 (7)
	Serum potassium and phosphorus concentrations	78 (44)

Of the 7 participants who answered an end-of-study usability survey, all were motivated by the sense of security derived from using the system (Table 3). The devices, including smartphone, did not cause physical discomfort to any of the participants. Of the 7 patients, 6 (86%) felt that using the system helped to improve their lifestyle and dialysis self-management and that the system had a positive impact on their dialysis management. The average usage time per day was 7.7 (SD 3.9) minutes, and 6 of the 7 patients (86%) felt the system was worth the time they spent. Only one participant—who took 10 minutes per day—felt that using the system was unduly time-consuming. Of the 7 participants, 5 (71%) were willing to continue to use the system (Table 3), citing such reasons as the way it made self-management easier or motivated them to improve health status (Table 4). One (14%) expressed the desire to continue using it if an additional function could be implemented to enable management of blood pressure and medication, while another patient (14%) was undecided (Tables 3 and 4).

**Table 3:** Usability survey results.

Statement or question (n=7)	Response
I could use a smartphone with no problem. (n [%])	5 (71)
I could use a weight scale with no problem. (n [%])	6 (86)
The interface of the data registration was easy to use. (n [%])	7 (100)
The interface of the data browsing was easy to watch. (n [%])	6 (86)
The instructions were easy to understand. (n=5^a^) (n [%])	5 (100)
The devices caused me physical discomfort. (n [%])	0 (0)
I easily incorporated using the system into my daily routine. (n [%])	5 (71)
Using the system gave me a sense of security. (n [%])	7 (100)
Participation in the study helped me to improve lifestyle and dialysis self-management. (n [%])	6 (86)
Using the system caused me some problems. (n [%])	1(14)^b^
How much time did you spend using the system per day? (minutes, mean [SD])	7.7 (3.9)
Is the system worth using for the time you spent? (n [%])	6 (86)
Did this system give a positive impact on your dialysis management? (n [%])	6 (86)
Would you like to continue using this system? (n [%])	5 (71)^c^

^a^Two participants answered that they did not use the instructions.

^b^One participant felt that using the SMART-D was time-consuming.

^c^The reasons given by the participants are shown in Table 4.

**Table 4:** Reasons why the participants would like or not like to continue using the Self-Management and Recording System for Dialysis.

Would you like to continue using this system? (n=7)		
Yes (5)	No (1)	Undecided (1)
Since the water intake of the day can be confirmed at a glance, I can be careful of the water intake. It makes self- management easier. It helps me motivated to improve my health status. Recording the body weight every day enabled me to know the physical condition better. Getting aware that the data deviated from the normal range motivated me to make efforts to return to the target range. It is convenient because the change of data can be seen immediately.	I would like to continue using it if additional function is implemented to enable management of blood pressure and medication.	Although it is useful for monitoring water intake at a glance, it is sometimes too much work.

The medical staff in the dialysis facilities monitored the data transferred from each patient's module and found no reason to contact the patients outside of the regular dialysis sessions during the 2 weeks.

### Clinical Outcomes

For IWG, serum potassium concentrations, and serum phosphorus concentrations, the values during the study and follow-up periods were compared with those at baseline with no significant differences found in the SMART-D group and the non–SMART-D group (Multimedia Appendix 1).

### Quality of Life

The QOL before and after the study was evaluated, as noted, by the KDQOL scale, which consists of 79 items, 36 asking about health-related QOL in general (the Medical Outcomes Study SF-36) and 43 asking about QOL as it is affected by kidney disease and by dialysis. The scores range from 0 to 100, with higher scores indicating better QOL [19]. As noted in Methods, 8 patients in the SMART-D group and 10 in the non–SMART-D group completed the questionnaire before and after the study period.

The KDQOL scores of the SMART-D group were compared before and after the study period (Table 5). The Social Functioning score showed a significant change, improving by 9.4 points (mean values 75.0 to 84.4; *P*=.048), while the other KDQOL scores remained unchanged. In the non–SMART-D group, the Role Functioning Emotional score was lower than in the SMART-D group before the study period (Multimedia Appendix 2), and none of the KDQOL scores improved over time (Multimedia Appendix 3).

**Table 5:** Comparison of changes in the Kidney Disease Quality of Life scores before and after the study period in the Self-Management and Recording System for Dialysis group (n=8).

Kidney Disease Quality of Life	Baseline score mean (SD)	Follow-up score mean (SD)	*P* value
Symptoms/problems	83.6 (7.8)	84.4 (8.4)	.84
Effect of kidney disease	78.4 (16.3)	79.7 (16.3)	.42
Burden of kidney disease	36.7 (21.8)	38.3 (16.5)	.79
Work status	75.0 (37.8)	75.0 (37.8)	>.99
Cognitive function	87.6 (11.8)	87.5 (12.6)	.52
Quality of social interaction	90.5 (10.1)	86.7 (19.2)	.20
Sleep	58.5 (7.5)	66.9 (18.1)	.16
Social support	68.7 (25.9)	62.5 (36.5)	.58
Dialysis staff encouragement	75.0 (11.6)	82.8 (14.8)	.10
Patient satisfaction	85.4 (16.5)	79.2 (21.3)	.20
Physical functioning	91.3 (7.4)	83.8 (20.0)	.28
Role functioning physical	78.1 (28.1)	84.4 (35.2)	.67
Bodily pain	65.3 (26.2)	74.1 (25.9)	.17
General health perception	52.9 (16.0)	54.4 (13.5)	.65
Vitality	56.3 (15.8)	65.0 (22.7)	.10
Social functioning	75.0 (20.0)	84.4 (14.6)	.048
Role functioning emotional	91.7 (15.4)	100 (0.0)	.17
Mental health	75.0 (16.1)	74.6 (16.)2	.96

## Discussion

### Principal Findings

This study has shown the feasibility and usability of SMART-D, a novel smartphone-based self-management system for dialysis patients. Patients record their body weight and predialysis serum potassium and phosphorus concentrations, and the app displays the data in graph form showing automatic evaluation with reference to target values, providing the patients with visual feedback and reinforcement that helps self-monitoring.

The average completion rates for body weight, predialysis/postdialysis weight, and serum potassium and phosphorus concentrations were high: respectively, 89% (SD 23), 95% (SD 7), and 78% (SD 44) (Table 2), proving that SMART-D is a feasible tool. The usability survey demonstrated that participants were comfortable with the use of the system, and most of the participants reported that using the system helped improve their lifestyle and dialysis self-managements and that they wanted to continue using the system (Tables 3 and 4).

The KDQOL scores of the SMART-D group’s Social Functioning score improved significantly while their other KDQOL scores remained unchanged (Table 5). This suggests that SMART-D may be effective in improving the QOL of dialysis patients. The clinical relevance of QOL in dialysis patients has been recognized since it was shown to be an independent predictor of mortality and hospitalization [11,12,21]. Improving self-management is an effective way to improve QOL in hemodialysis patients [13-15], and SMART-D may have contributed to improving QOL by supporting self-management. However, this result should be interpreted with caution because the current study was not randomized and had a small sample size with a short study period.

### Self-Monitoring and Dialysis

More and more evidence has recently emerged that smartphone-based apps can be powerful tools in supporting self-management for patients with chronic conditions. We have reported on DialBetics, a smartphone-based self-management support system for diabetes patients. DialBetics is an interactive system that provides real-time advice on diet and lifestyle based on input of patient data measured at home including exercise and diet. A 3-month randomized study showed that DialBetics is an effective tool for improving glycemic control [16]. Compared with self-management systems such as DialBetics, SMART-D is a relatively simple system with limited feedback. The system provides no real-time lifestyle advice except when medical staff considers it necessary to intervene with the patients. Rather, the function of SMART-D is to support patient self-monitoring, making it easier and more sustainable for them.

Evidence has suggested that simple self-monitoring is effective for managing various conditions. Obesity is one of them. It was reported that frequent self-weighing by obese patients, accompanied by daily charting of their weight pattern, was effective in weight reduction programs [22,23]. It is interesting and highly relevant that, while increasing patient nutritional education does not necessarily help patients modify behavior related to obesity in diverse community settings [24], self-weighing—a simple self-monitoring behavior—along with behavioral weight-loss education has proven effective. The power of self-monitoring is quite understandable. With obesity, for instance, self-monitoring promotes greater awareness of how behaviors such as diet and physical activity are impacting weight. Frequent self-weighing provides immediate feedback, helping patients perceive the connection between weight changes and their specific eating behavior and/or lifestyles, making it easier for them to correct these behaviors. Patients who adhere to diets are visibly rewarded by weight loss, and this in turn reinforces adherence to diet and lifestyle changes and, thus, maintenance of weight reduction. Pacanowski and Levitsky [25] reported that frequent self-weighing with visual feedback of weight history—even without any prescribed diet or exercise plan—was effective enough to produce and maintain weight loss. Such is the importance of self-monitoring!

The relevance to hemodialysis is clear. As with obese patients, eating behavior and lifestyle directly impact the body weight and serum potassium and phosphorus concentrations of dialysis patients. Despite repeated attempts to educate such patients about the consequences of noncompliance, their adherence to fluid control and diets that restrict sodium, potassium, and phosphorus intake is generally poor. The SMART-D system helps these patients self-monitor by making it easy for them to view their history of body weight and serum potassium and phosphorus levels with reference to target values. This self-monitoring should help the patients attribute body weight and blood test results to their diet and lifestyle behavior, correct errant behavior, and improve their self-management skills. Medical staff members will also benefit from SMART-D because the system lets them monitor the course of patient at-home body weight and gives them an electronically accurate, objective view of how faithfully patients are measuring their weight and checking their laboratory data, an important insight into patient attitude and seriousness about improving their health status. Combined with access to their own data from the laboratory, this additional information enables medical staff to give patients more precise and appropriate advice on diet and lifestyle.

### Limitations

There are several limitations of this study. First, the study period was too short to observe improvement of clinical outcomes although SMART-D is expected to improve clinical outcomes and QOL by supporting self-management; previous studies have reported that apps and programs to improve adherence of dialysis patients to diet and lifestyle changes led to improvement of clinical outcomes and QOL [17,26,27]. Since our study period of 2 weeks was markedly shorter than those of the studies reporting such notable effects, a longer period is necessary to assess the impact of SMART-D on clinical outcomes and QOL. However, we assumed that feasibility and usability could be sufficiently evaluated in a 2-week study period because we had previously successfully evaluated usability and compliance of a smartphone-based self-management tool for type 2 diabetes patients in a 1-week study [28]. Others have also demonstrated the feasibility and acceptability of a mobile phone-based self-management tool for asthma patients in a 2-week study [29]. Even so, a longer study is necessary because it has been reported that the longer a study period becomes, the greater the decline in completion rates [16,30-32].

Second, the overall favorable results of the usability survey might be biased because only those patients willing to use SMART-D were assigned to the SMART-D group while the patients assigned to the non–SMART-D group did not wish to use the system. The differences of demographic characteristics between the two groups are likely to reflect the patient characteristics associated with willingness to use such tools. Patients in the SMART-D group tended to be younger and more likely to be users of a smartphone, PDA, and the Internet (Table 1), suggesting that patients in the SMART-D group had better technology literacy. Future tasks include developing more user-friendly systems that can be widely used by patients with lower information and communication technology literacy.

### Conclusions

We have shown the feasibility of SMART-D, a novel smartphone-based self-management system for hemodialysis patients. The system supports self-monitoring of three crucial mortality-related factors that patients can modify by correcting their diet and lifestyle: IWG and predialysis serum potassium and phosphorus concentrations. Most of the participants reported that using SMART-D helped improve their self-management. Further large-scale study with a larger cohort and longer system use and follow-up periods is needed to evaluate the effects of SMART-D on clinical outcomes and QOL.

## Appendix

Multimedia Appendix 1 Table showing clinical parameters at baseline, study period and follow-up period in the Self-Management and Recording System for Dialysis group (n=9) and the non-Self-Management and Recording System for Dialysis group (n=11).

Multimedia Appendix 2 Table showing comparison of Kidney Disease Quality of Life scores between the Self-Management and Recording System for Dialysis group and the non-Self-Management and Recording System for Dialysis group before the study period.

Multimedia Appendix 3 Table showing comparison of changes in Kidney Disease Quality of Life scores before and after the study period in the non-Self-Management and Recording System for Dialysis group.

## Abbreviations

CSV: comma-separated values
DW: dry weight
ICT: information and communication technology
IWG: interdialytic weight gain
JSDT: Japanese Society for Dialysis Therapy
KDQOL: Kidney Disease Quality of Life
KDQOL-SF: Kidney Disease Quality of Life Short Form
PDA: portable digital assistant
QOL: quality of life
SMART-D: Self-Management and Recording System for Dialysis
