# Supplementary material for: Testing the Feasibility and Usability of a Novel Smartphone-Based Self-Management Support System for Dialysis Patients: A Pilot Study
Source: JMIR Res Protoc. 2017 Apr 20;6(4):e63. doi: 10.2196/resprot.7105 (PMC5418525; doi:10.2196/resprot.7105)
Supplement: Multimedia Appendix 1 [file resprot_v6i4e63_app1.pdf]

Multimedia Appendix 1 Table showing clinical parameters at baseline, study period and follow-up period in the Self-Management and Recording System for Dialysis group (n=9) and the non-Self-Management and Recording System for Dialysis group (n=11).

	Baseline	Study period	Follow-up period	
**SMART-D[a] group (n=9)**	mean (SD)			*P* value
IWG[b] (%DW[c]/day)	2.2 (0.4)	2.1 (0.3)	2.1 (0.4)	.68
Serum potassium concentrations (mEq/L)	4.9 (0.6)	5.1 (0.7)	5.0 (0.5)	.07
Serum phosphorus concentrations (mg/dL)	5.4 (1.2)	5.2 (1.0)	5.2 (1.2)	.67
**Non-SMART-D group (n=11)**	mean (SD)			*P* value
IWG (%DW/day)	2.2 (0.4)	2.3 (0.5)	2.4 (0.5)	.13
Serum potassium concentrations (mEq/L)	5.1 (0.8)	5.1 (0.6)	5.1 (0.7)	.84
Serum phosphorus concentrations (mg/dL)	5.0 (1.1)	5.0 (1.3)	5.2 (1.0)	.73

[a]SMART-D: Self-Management and Recording System for Dialysis.

[b]IWG: interdialysis weight gain.

[c]DW: dry weight.
