# Supplementary material for: Testing the Feasibility and Usability of a Novel Smartphone-Based Self-Management Support System for Dialysis Patients: A Pilot Study
Source: JMIR Res Protoc. 2017 Apr 20;6(4):e63. doi: 10.2196/resprot.7105 (PMC5418525; doi:10.2196/resprot.7105)
Supplement: Multimedia Appendix 2 [file resprot_v6i4e63_app2.pdf]

Multimedia Appendix 2 Table showing comparison of Kidney Disease Quality of Life scores between the Self-Management and Recording System for Dialysis group and the non-Self-Management and Recording System for Dialysis group before the study period.

	SMART-D[a] (n=8)	Non SMART-D (n=10)	
Kidney Disease Quality of Life	Scores, Mean (SD)		*P* value
Symptoms/Problems	83.6 (7.8)	80.5 (16.2)	.93
Effect of kidney disease	78.4 (16.3)	73.1 (18.6)	.54
Burden of kidney disease	36.7 (21.8)	36.1 (27.6)	.96
Work status	75.0 (37.8)	45.0 (49.7)	.21
Cognitive function	87.6 (11.8)	85.7 (18.6)	.91
Quality of social interaction	90.5 (10.1)	76.7 (21.1)	.09
Sleep	58.5 (7.5)	54.0 (16.5)	.48
Social support	68.7 (25.9)	65.1 (31.9)	.80
Dialysis staff encouragement	75.0 (11.6)	75.0 (12.5)	1.00
Patient satisfaction	85.4 (16.5)	84.4 (17.0)	.96
Physical Functioning	91.3 (7.4)	79.5 (14.8)	.06
Role Functioning Physical	78.1 (28.1)	61.1 (39.7)	.39
Bodily Pain	65.3 (26.2)	67.0 (28.8)	.90
General Health Perception	52.9 (16.0)	58.1 (8.5)	.39
Vitality	56.3 (15.8)	69.0 (13.3)	.08
Social Functioning	75.0 (20.0)	66.3 (14.5)	.30
Role Functioning Emotional	91.7 (15.4)	59.3 (40.1)	.046
Mental Health	75.0 (16.1)	80.3 (16.4)	.35

[a]SMART-D: Self-Management and Recording System for Dialysis.
