# Supplementary material for: Testing the Feasibility and Usability of a Novel Smartphone-Based Self-Management Support System for Dialysis Patients: A Pilot Study
Source: JMIR Res Protoc. 2017 Apr 20;6(4):e63. doi: 10.2196/resprot.7105 (PMC5418525; doi:10.2196/resprot.7105)
Supplement: Multimedia Appendix 3 [file resprot_v6i4e63_app3.pdf]

Multimedia Appendix 3 Table showing comparison of changes in Kidney Disease Quality of Life scores before and after the study period in the non-Self-Management and Recording System for Dialysis group.

	Non-SMART-D[a] group (n=10)		
Kidney Disease Quality of Life	Baseline	Follow-up	
	Scores, Mean (SD)		*P* value
Symptoms/Problems	80.5 (16.2)	77.3 (19.9)	.06
Effect of kidney disease	73.1 (18.6)	68.7 (20.6)	.03
Burden of kidney disease	36.1 (27.6)	27.5 (18.7)	.08
Work status	45.0 (49.7)	55.0 (49.7)	.17
Cognitive function	85.7 (18.6)	90.0 (14.1)	.30
Quality of social interaction	76.7 (21.1)	82.7 (13.4)	.49
Sleep	54.0 (16.5)	54.6 (14.7)	.89
Social support	65.1 (31.9)	60.0 (34.4)	.59
Dialysis staff encouragement	75.0 (12.5)	52.8 (41.8)	.12
Patient satisfaction	84.4 (17.0)	72.2 (23.6)	.21
Physical Functioning	79.5 (14.8)	66.3 (22.6)	.06
Role Functioning Physical	61.1 (39.7)	67.5 (37.4)	.38
Bodily Pain	67.0 (28.8)	62.3 (28.0)	.61
General Health Perception	58.1 (8.5)	51.5 (11.6)	.04
Vitality	69.0 (13.3)	59.5 (16.4)	.04
Social Functioning	66.3 (14.5)	64.4 (21.7)	.79
Role Functioning Emotional	59.3 (40.1)	66.7 (41.6)	.23
Mental Health	80.3 (16.4)	69.6 (16.2)	.07

[a]SMART-D: Self-Management and Recording System for Dialysis.
